# Subtype-Independent ANP32E Reduction During Breast Cancer Progression in Accordance with Chromatin Relaxation

**DOI:** 10.1186/s12885-021-09077-9

**Published:** 2021-12-18

**Authors:** Garrett L. Ruff, Kristin E. Murphy, Zachary R. Smith, Paula M. Vertino, Patrick J. Murphy

**Affiliations:** 1 Department of Biomedical Genetics, University of Rochester Medical Center, Rochester, NY 14642 USA; 2 Wilmot Cancer Institute, University of Rochester Medical Center, 601 Elmwood Avenue, Rochester, NY 14624 USA

**Keywords:** Chromatin, Epigenetics, Breast Cancer, FOXA1, ANP32E, Bioinformatics

## Abstract

**Background:**

Chromatin state provides a clear decipherable blueprint for maintenance of transcriptional patterns, exemplifying a mitotically stable form of cellular programming in dividing cells. In this regard, genomic studies of chromatin states within cancerous tissues have the potential to uncover novel aspects of tumor biology and unique mechanisms associated with disease phenotypes and outcomes. The degree to which chromatin state differences occur in accordance with breast cancer features has not been established.

**Methods:**

We applied a series of unsupervised computational methods to identify chromatin and molecular differences associated with discrete physiologies across human breast cancer tumors.

**Results:**

Chromatin patterns alone are capable of stratifying tumors in association with cancer subtype and disease progression. Major differences occur at DNA motifs for the transcription factor FOXA1, in hormone receptor-positive tumors, and motifs for SOX9 in Basal-like tumors. We find that one potential driver of this effect, the histone chaperone *ANP32E,* is inversely correlated with tumor progression and relaxation of chromatin at FOXA1 binding sites. Tumors with high levels of *ANP32E* exhibit an immune response and proliferative gene expression signature, whereas tumors with low *ANP32E* levels appear programmed for differentiation.

**Conclusions:**

Our results indicate that ANP32E may function through chromatin state regulation to control breast cancer differentiation and tumor plasticity. This study sets a precedent for future computational studies of chromatin changes in carcinogenesis.

**Supplementary Information:**

The online version contains supplementary material available at 10.1186/s12885-021-09077-9.

## Background

Cellular programming is controlled by epigenetic modifications, transcription factor binding, and DNA packaging within the nucleus. These mechanisms regulate how gene transcription machinery gains access to DNA at transcription start sites and cis-regulatory enhancers, ultimately controlling cellular programming through regulation of gene expression. Regions with more accessible chromatin tend to be more highly transcribed, and inaccessible regions are typically silent [1]. Overall, chromatin accessibility is generally stable in terminally differentiated cells, along with steady gene expression profiles, and the majority of chromatin state dynamics occur either during embryonic development or as a consequence of disease progression, including during carcinogenesis [1–3]. Breast cancer is among the most frequent and well-studied forms of cancer worldwide, but chromatin state specific differences among breast cancers have not been established.

Breast cancer represents the most diagnosed cancer in women [4] with an estimated 2.1 million newly diagnosed cases globally in 2018 [5]. Measurements of chromatin states have the potential to provide new insights into breast cancer mechanism and may ultimately lead to innovative therapy strategies. For example, a recent study of 410 tumors from The Cancer Genome Atlas (TCGA) used chromatin accessibility measurements to identify more than 500,000 putative gene regulatory elements, including thousands of genomic locations where accessibility differences occurred in a disease-specific and tissue-specific manner [2]. Separate studies of myeloma have also found that accessibility levels at gene-distal enhancer regions enable accurate prediction of nearby oncogene expression levels, as well as cancer subtype classification [6]. Similar breast cancer focused studies are lacking and have the potential to identify parallel associations.

Changes in transcription factor activity occur during breast carcinogenesis in a manner associated with discrete cancer outcomes. Increased transcription factor binding generally leads to increased expression of neighboring genes [1–3], and many factors that are normally active during development become reactivated in breast cancer to influence tumorigenic behavior. For example, SOX9 and FOXC1 are important for developmental regulation of transcription in multipotent neural crest stem cells [7, 8], and they become reactivated in breast cancer to co-regulate Basal-like cancer initiation and proliferation [9]. FOXA1, which is normally active in hematopoietic progenitor cells, acts coordinately with ER to suppress Basal-like programming and reinforce the luminal phenotype [10, 11]. Furthermore, hyperactivity of FOXA1 promotes pro-metastatic transcriptional programs in endocrine-resistant tumors [12, 13]. These transcription factors are able to bind DNA most effectively at accessible chromatin locations, and most factors are non-functional at inaccessible binding sites [1]. Thus, assessing chromatin accessibility in breast cancer tumors at specific transcription factor binding sites could be highly informative for studying the molecular function of numerous factors during carcinogenesis.

We recently defined the histone chaperone protein ANP32E as a genome-wide regulator of chromatin accessibility in mouse fibroblasts [14]. ANP32E functions to modulate the installation/removal of H2A.Z from chromatin, regulating chromatin remodeler activity and limiting chromatin accessibility. We found that loss of ANP32E caused thousands of gene promoters and enhancers to become more “open”, leading to activation of neighboring genes. These changes were accompanied by cellular reprogramming events where loss of ANP32E caused cells to take on a more differentiated transcriptome phenotype. Interestingly, a recent study suggests that ANP32E may be an independent prognostic marker for human breast cancers, where higher ANP32E protein levels are associated with the TNBC subtype and correlated with a shorter overall and disease-free survival. Moreover, forced downregulation of ANP32E suppressed TNBC tumor growth in xenograft models [15]. However, the precise mechanisms by which ANP32E functions to support breast cancer growth and its role in defining breast cancer phenotypes has not been fully established.

To gain insight into chromatin state function and heterogeneity in human breast cancer, we used an unsupervised computational approach to segregate tumors into defined groups based solely on genome-wide chromatin accessibility patterns. Basal-like tumors segregated as a homogeneous class within group 1, whereas a mixture of tumor types was found within group 2, including nearly all Luminal-B and HER2-enriched tumors, and group 3 consisted primarily of lobular Luminal-A tumors. By defining the chromatin accessibility ‘signature’ associated with each group, we identified DNA sequence motifs for specific transcription factors. SOX9 motifs were most accessible in group 1 tumors, and FOXA1 motifs were most accessible in hormone receptor positive tumors within groups 2 and 3. Finally, we found that expression for the chromatin factor ANP32E was anti-correlated with tumor progression and with accessibility at FOXA1 binding sites among group 2 and 3 tumors, suggestive of a novel mechanism by which FOXA1 activity may be regulated in breast cancer tumors. Our results highlight the potential for future disease focused studies of chromatin accessibility, as well as epigenetic therapies directed at disrupting chromatin regulatory factors.

## Methods

### Measurements of Chromatin Accessibility, Gene Expression and Classification of Tumors

Datasets from the assay for transposase-accessible chromatin followed by sequencing (ATAC-Seq) were downloaded from TCGA-BRCA project in the National Cancer Institute’s (NCI) Genomic Data Commons (GDC) [16]. Datasets were downloaded as bam files, sorted, and read count normalized with DeepTools (v3.1.3) (bamCoverage -bs 10 -rpkm) [17]. MACS2 (v2.1.4) was used for peak calls (bdgpeakcall -c 35 -g 100 -l 100) [18]. A union peak set was generated containing all peaks across datasets (n=245133), and accessibility in these regions was scored for all tumors. Gene expression datasets were also downloaded from the TCGA-BRCA project. Files were downloaded as tables and matched to ATAC-Seq with Case ID. All 1222 expression files available in the TCGA-BRCA project were also combined into a union expression table. Tumor stage and IHC subtype were extracted from the TCGA-BRCA project in the NCI’s GDC. PAM50 subtype [19], histological subtype [20], and general patient demographics [20] data were obtained in cBioPortal [21, 22].

### CUT&Tag Sequencing

MCF-7 cells were cultured according to methods described previously [23]. To measure genomic ANP32E enrichment, CUT&Tag experiments were performed as previously described [14, 24], using an antibody recognizing ANP32E (Thermo PA5-42860). Libraries were sequenced on Illumina NextSeq550 in 75bp paired-end mode and raw sequencing data was aligned to Hg38 using Bowtie2 [25]. Peak calling was performed with MACS2 bdgpeakcall (-g 100 -l 100 -c 90) and read count normalization was performed using DeepTools (v3.1.3) (bamCoverage -bs 10 -RPKM). ENCODE ChIP-Seq datasets for FOXA1 were handled similarly with the exception of peak thresholds (bdgpeakcall -c 10).

### Unsupervised Dimensional Reduction and Clustering

The union peak table (described above) was uploaded in R and scores were normalized by ranking regions from minimum to maximum accessibility for each tumor. This table was then input into UMAP package (n_neighbors=10) [26]. UMAP output three tumor groups by agnostically grouping tumors based on similarities in chromatin accessibility patterns. To identify regions where accessibility differences occurred, log2 fold change (log2FC) values were calculated from a region’s average accessibility within a tumor group compared with its accessibility in all other tumors. Signatures 1, 2 and 3 consisted of regions with a log2FC greater than 2.5 for groups 1, 2 and 3, respectively. Tumors were considered individually rather than as replicates, and therefore significance measurements were not assessed in defining divergent accessibility or gene expression groups.

### Data Visualization

The pheatmap package in R was used to create heatmaps of chromatin accessibility and gene expression, annotated by tumor characteristics. The ggplot2 package was used to create scatterplots and superimpose characteristics, such as cancer type, on UMAP plots. Integrative Genomics Viewer (IGV) [27] was used to visualize chromatin accessibility in tumor groups and stages. DeepTools (computematrix and plotheatmap) was used to create heatmaps of accessibility and ChIP-Seq binding across regions. The Hg38 genome assembly was used.

### Annotation of Chromatin Signatures and Gene Ontology Analyses

HOMER (v4.10) was used to annotate and find motifs enriched in each chromatin signature (see above) [28], and group accessibility trends at those motifs were subsequently determined. Gene ontologies for chromatin regions were determined with GREAT, which associates regions to any gene whose transcription start site is within 1000 kb [29]. Gene ontologies for genes from divergent gene expression analyses were determined with Enrichr [30, 31].

### Dataset Availability

ENCODE was used to download ChIP-Seq data from the MCF-7 cell line for FOXA1 (ENCSR126YEB), H3K27ac (ENCSR752UOD), H2A.Z (ENCSR057MWG) and ER (ENCSR463GOT) [32, 33]. BigWig files of log2FC over control were downloaded from the ENCODE portal with the following identifiers: ENCFF795BHZ (FOXA1), ENCFF063VLJ (H3K27ac), ENCFF589PLM (H2A.Z), and ENCFF237WTX (ER). ATAC-Seq data from TNBC cell-lines were acquired using GEO accession GSE129646 [34]. MCF-7 CUT&Tag data for ANP32E are available using GEO accession GSE188942.

### GSEA

Using the union expression dataset, tables of tumors in the top and bottom decile of *ANP32E* expression were generated. In order to associate gene ontologies with *ANP32E* expression, the average gene expressions of the top and bottom deciles were input into GSEA, which then converted normalized counts data to ranked lists for enrichment scoring [35, 36]. To isolate this effect from ANP32E’s association with Basal-like tumors, we sought to eliminate the Basal-like subtype. Using expression of *FOXA1* and *GATA3*, two PAM50 markers, we removed the tumors that were in the bottom quartile of expression for both genes. Testing this method on the 74 known tumors, this results in 14 tumors being eliminated. 10 of the 12 known Basal-like tumors were removed, and 12 of the 14 tumors removed were in group 1. Since this method was shown to be effective in removing the majority of Basal-like tumors from the sample, we applied it to all tumors in the TCGA-BRCA project. This resulted in removing 112 of the 1222 expression files available. We then repeated the GSEA analysis with this subset of tumors.

Statistical analyses were done with R statistical software (v3.6.3), and p-values obtained are from parametric t-tests. Log2 fold-change values were calculated with a pseudo-count of 1.

## Results

### Patterns of chromatin accessibility segregate breast tumors into distinct subtypes

**Fig. 1:**
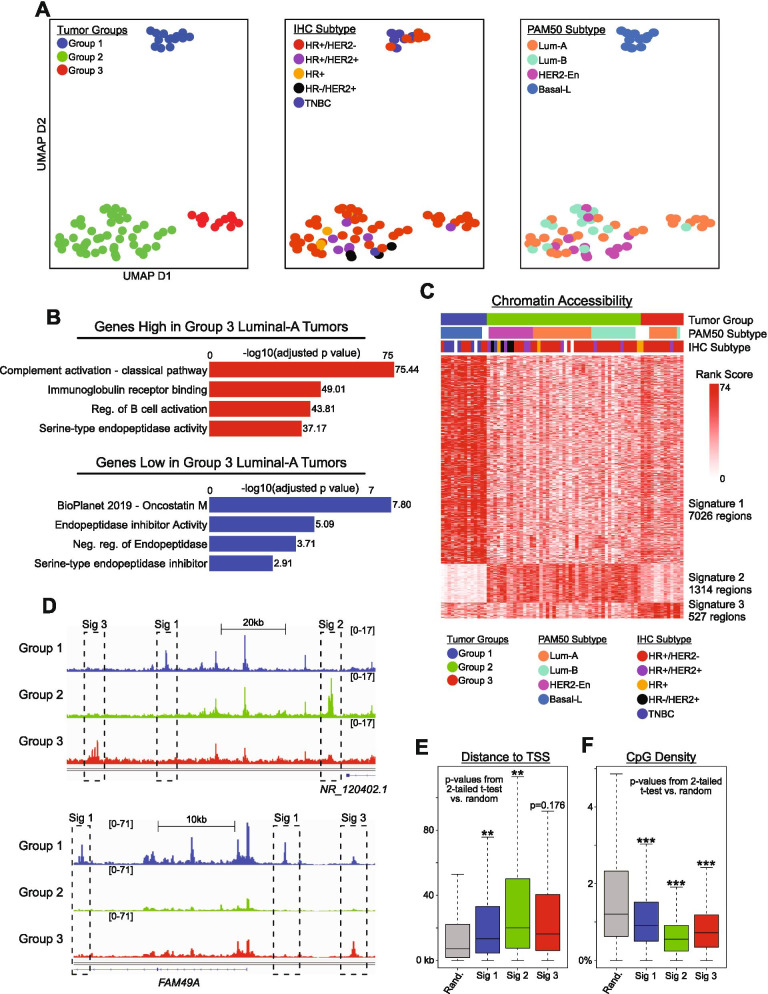
Chromatin Accessibility Distinguishes Breast Cancer Subtypes. A) UMAP dimension reduction plots depicting three distinct groups of tumors, colored by group (n=74), IHC subtype (n=69) and PAM50 subtype (n=65). B) Bar charts depicting significance of gene ontology results from Enrichr, investigating genes found to have higher and lower expression in Luminal-A tumors in group 3 compared to group 2. Adjusted p-values obtained within Enrichr. C) Heatmap showing 3 groups of chromatin regions, each showing greater accessibility in their respective tumor group compared to the rest (Lg2FC > 2.5). D) Screenshots from IGV depicting average accessibility of tumor groups in regions within each chromatin signature. E-F) Boxplots comparing chromatin signatures by regions’ distance to transcription start sites (E) and CpG density (F), with random accessible regions from the genome as a control. P-values obtained from two-tailed parametric t-tests. * is p<0.01, ** is p<0.001, *** is p<0.0001

Chromatin accessibility has been used for defining cell identities, for establishing tissues of origin, and for measuring developmental cell-state transitions [37–40]. We therefore sought to measure chromatin differences in breast tumors, but rather than simply comparing between established subtypes, we opted for an unsupervised approach, enabling both independent corroboration of established mechanisms, and the potential for uncovering new inferences. DNA sequence data from ATAC-Seq of 74 primary invasive breast carcinomas were acquired from the TCGA-BRCA project [2, 41], and datasets were normalized based on total mapped reads. Enrichment scores at ‘peaks’, representing high accessibility regions [18] (245133 union peaks), were then assessed using Uniform Manifold Approximation and Projection (UMAP) [26], wherein tumors segregated into three distinct groups (Fig. 1A), with no obvious differences in demographics between groups (Fig. S1A & S1B). Most chromatin differences occurred along UMAP dimension 2, where tumors within group 1 bore the greatest distinction from groups 2 and 3 (Fig. 1A - left).

We next assessed how this chromatin-based grouping corresponded with established breast cancer subtypes, expecting that chromatin patterns would be somewhat associated with gene expression-based classification – based on the established relationships between chromatin accessibility and transcriptional regulation [1]. Measurements of protein levels for estrogen receptor (ER), progesterone receptor (PR), and HER2 (gene/protein), were previously used to classify tumors as hormone receptor positive (HR+) or negative (HR-), and HER2 positive (+) or negative (-). Triple-negative breast cancers (TNBC) were classified as those lacking expression of all these biomarkers [42]. PAM50 (Prediction Analysis of Microarray 50) classification was also applied, relying on gene expression profiling to identify “intrinsic subtypes”, as Luminal A (Lum-A), Luminal B (Lum-B), HER2-enriched, and Basal-like (Basal-L) [43]. Indeed, our chromatin-based UMAP grouping corresponded better with PAM50 subtypes than with protein-based immunohistochemistry (IHC) subtypes (Fig. 1A). While HR+/HER2- tumors were distributed across all three groups, Basal-L tumors were found exclusively within group 1, and nearly all HER2-enriched and Lum-B tumors were within group 2 (Fig. S1C & S1D). Interestingly, a subset of Lum-A tumors were classified as a distinct set of tumors within group 3 (Fig. 1A - right) (analyzed subsequently). As expected, given the enrichment of TNBC/Basal-L tumors in group 1, mutations in *TP53* were over-represented in group 1, whereas mutations in *PIK3CA, GATA3* and *CDH1* were underrepresented (Fig. S1E). Also as expected, given the relationship to PAM50 subtype, expression of *FZD7*, *SOX9*, and *MYC* was higher for tumors within group 1, whereas tumors in group 2 and 3 had higher expression of *FOXA1* and *GATA3* (Fig. S1F).

Based on the success of these initial proof-of-principle measurements, we next investigated whether differences in chromatin patterns corresponded with unique tumor behaviors or novel underlying biological differences. In this regard, two tumor classes stood out. HR+/HER2- tumors, which were distributed across all three groups, and Lum-A tumors, which were split between groups 2 and 3. To assess differences in cellular programing for these tumors, we measured differences in the mean transcriptome patterns for each class (Fig. S1G & S1H) and used gene ontology (GO) analysis to identify molecular pathways or pathologies associated with transcriptome differences. Genes involved in hormone signaling tended to be under-expressed in the HR+/HER2- tumors in group 1 compared with similarly classified tumors from other groups (Fig. S2A), including *ESR1*, *PR*, *ERBB2*, and *AR* (Fig. S2B), suggesting that the Basal-L class of HR+/HER2- tumors were more similar to TNBC tumors than non-Basal-L HR+/HER2- tumors. For the subset of Lum-A tumors (8 of 24) classified as group 3, there was no apparent difference in the expression of the classic biomarker genes (*ESR1*, *PR*, *ERBB2*) or AR (Fig. S2C), but transcriptome differences largely reflected dysregulation of genes involves in humoral immune response and inflammatory pathways (which were enriched and depleted) respectively in Lum-A tumors within group 3 (Fig. 1B). Taken together, these data suggest that the chromatin state differences in breast cancer largely occur in Basal-L tumors (as compared with non-Basal-L tumors) and within a distinct subset of Lum-A tumors, potentially resulting from immune evasion [44].

To gain further insight into the factors driving group classification, we identified the genomic regions where high levels of accessibility were present for tumors within each respective group, as compared with all other tumors (Log2FC>2.5). This enabled us to define a set of accessible loci (signature regions) which independently partitioned tumors in a manner nearly identical to UMAP grouping (Fig. 1C & D). Interestingly, the signature sites for all 3 groups tended to be further away from the nearest annotated TSS (Fig. 1E) and less CpG rich (Fig. 1F), as compared with randomly-selected accessible peak regions, suggesting that they might represent distal regulatory elements or enhancers. GREAT analysis [29] (identifying all genes within 1000 kb) revealed that genes near signature 1 sites were involved in exocrine gland development, consistent with these tumors arising from the basal layer of mammary exocrine glands, and signature 2 sites were located nearest to hormone responsive genes, consistent with the abundance of HR+ and Lum-A/B tumors in this group (Fig. S2D). By contrast, genes associated with signature 3 sites were enriched in functions involved in cell metabolism, suggesting that a unique metabolic program may distinguish tumors in this group from those that otherwise bear a Lum-A gene expression signature. Applying a similar approach to Lum-A tumors within group 2 versus group 3, as noted previously, we found that regions of higher accessibility in group 2 Lum-A tumors were annotated to genes involved in development and morphogenesis, whereas regions with greater accessibility in group 3 were annotated to genes involved in carbohydrate metabolism (Fig. S2E & S2F). Both the subset of Lum-A tumors in group 3, and group 3 tumors in general, were distinguished by features associated with immune (Fig. 1B) and metabolic regulation (Fig. S2F), similar to gene expression characteristics for tumors previously identified as Lum-A invasive lobular carcinomas (ILC) [45–47]. Indeed, overlaying the tumor histology information (from the TCGA metadata) with UMAP classification indicated that ILC was over-represented in group 3 (Fig. S2G), and *CDH1* mutations, which are common in ILC tumors, were also found to be somewhat overrepresented (Fig. S1E). Notably however, this over-representation of ILC was not absolute, and several tumor samples within group 3 did not contain mutations in *CDH1*, suggesting that chromatin state differences may contribute independently to the transcriptome differences within this class.

Taken together, these results provide strong evidence that chromatin differences occur in association with particular cancer phenotypes, including IHC status and intrinsic subtype, and demonstrate that unsupervised chromatin state-based computational approaches are capable of independently distinguishing tumors in a manner well aligned with known features of breast cancer. These results also suggest that chromatin differences may be a more accurate reflection of tumor phenotype as compared with IHC status, and that differences in classification may reflect heterogeneity of HR protein expression within HR+ tumors, or variation in how (low vs. no) HR protein expression is stratified by different sites and pathologists. These conclusions prompted us to further characterize the chromatin signature regions which distinguish tumor groups, with the intent to uncover novel molecular aspects of tumor biology.

### Accessibility at FOX motifs is associated with cancer progression.

**Fig. 2:**
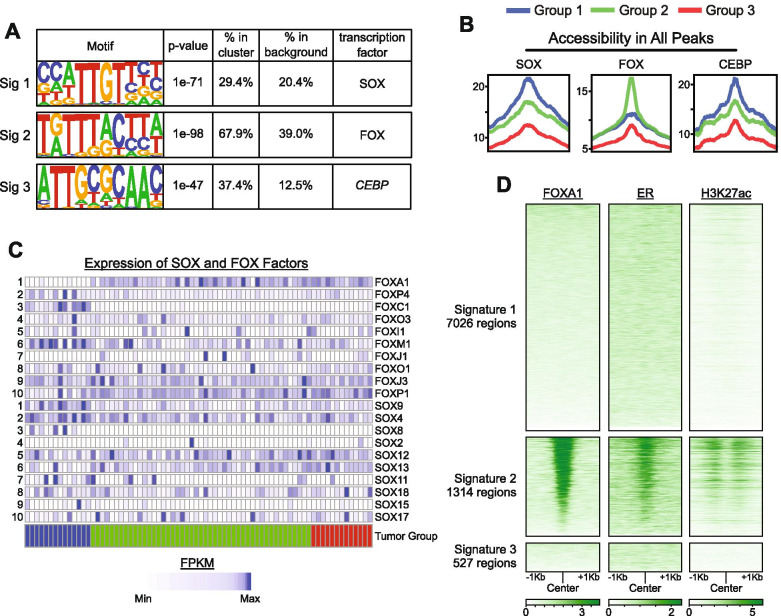
Accessibility at FOX and SOX Binding Sites Define Tumor Groups. A) Table displaying top motif result from HOMER for each chromatin signature. Due to similarity across SOX and FOX binding motifs, we refer to SOX6 simply as SOX, and FOXM1 simply as FOX. P-values obtained within HOMER. B) Profile plots depicting average accessibility of tumor groups in motif regions across all accessible peak regions in tumors, indicating that group 1 and 2 tumors show increased accessibility at SOX and FOX motifs, respectively. We again use the SOX6 motif to represent SOX motifs, and the FOXM1 motif to represent FOX motifs. Regions with no signal were ignored in the calculation of average accessibility. C) Heatmap showing expression of SOX and FOX factors across tumor groups. Factors are ordered from 1 to 10 by standard deviation across tumors. D) Heatmaps showing binding of FOXA1, ER, and H3K27ac in MCF-7 cells within regions from signatures 1, 2 and 3. Data from ChIP-Seq of MCF-7 cells; regions sorted from greatest to least FOXA1 enrichment.

To investigate how chromatin changes might contribute to biologically distinct tumor properties, we next investigated the genomic context of the established signature regions. The gene-distal nature of these signature regions (Fig. 1E) suggests that they might function as intergenic regulatory sites. In support of this possibility, sets of enriched DNA sequence motifs were identified (using HOMER) [28] within each signature region (Supplemental Table 1), as compared with background regions (consisting of 5000 randomly selected, similarly sized genome-wide accessible sites). SOX factor binding motifs were most enriched in signature 1 regions, FOX factor motifs were the most enriched in signature 2 regions, and CEBP motifs were the most enriched in signature 3 regions (Fig. 2A). Mapping of motifs within signatures 1, 2, and 3, revealed that accessibility differences occurred directly over motif locations (Fig. 2B). SOX motifs and CEBP motifs were most accessible in group 1 tumors, FOX motifs were most accessible in group 2 tumors, and interestingly, all three motifs were least accessible in group 3 tumors, suggesting that additional factors may underlie accessibility differences within this group. We next assessed levels of gene expression to determine which among the FOX and SOX family transcription factors might be involved. Here we found that group 1 tumors tended to express high levels of *SOX9*, *FOXC1*, and *FOXM1,* relative to tumors in groups 2 and 3, whereas group 2 tumors expressed high levels of *FOXA1* (Fig. 2C & S3A). Prior studies indicate that FOXA1 functions in conjunction with ER to influence enhancer activity and promote pro-metastatic transcriptional programming in breast cancer cell lines [12, 13]. We therefore investigated the binding patterns for these transcription factors at the defined signature regions. Indeed, chromatin immunoprecipitation data from MCF-7 cells [32, 33] revealed that FOXA1, ER, and H3K27ac (a marker of active enhancers) were enriched at signature 2 regions (Fig. 2D). Additionally, accessibility at FOX motifs tended to be lower for Lum-A tumors in group 3 (which were enriched for ILCs) as compared with Basal-L tumors or similarly classified tumors in group 2 (Fig. S3B), further supporting the premise that increased FOXA1 binding may functionally distinguish group 2 tumors from all other samples.

**Fig. 3:**
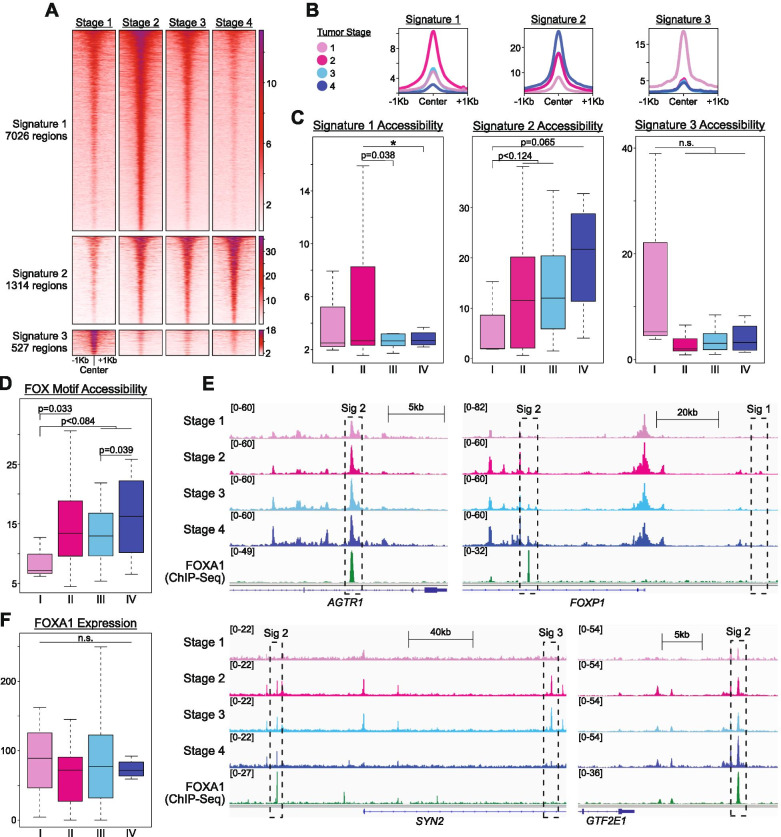
Chromatin Accessibility in FOX motifs and Signature 2 Regions Associate with Tumor Progression Stages. A-B) Heatmaps (A) and profile plots (B) showing accessibility in signatures 1, 2 and 3 across tumor stages. Heatmaps have regions ordered from greatest to least average accessibility across tumor stages, regions with no signal are ignored in calculation of average accessibility. C) Boxplots of accessibility in signatures 1, 2 and 3 across tumor stages, indicating that only signature 2 shows an accessibility trend across stages. D) Boxplot comparing accessibility of FOX motifs in accessible peak regions (n=96280) by tumor stage. E) Screenshots from IGV depicting average accessibility of tumor stages and FOXA1 binding in MCF-7 cells from ChIP-Seq in regions within each chromatin signature. ChIP-Seq data is Log2FC over control. F) Boxplot comparing FOXA1 expression levels across tumor stages. P-values in C, D and F obtained from one-tailed parametric t-tests. * is p<0.01, ** is p<0.001, *** is p<0.0001.

Activity of FOXA1 and ER transcription factors is known to promote pro-metastatic outcomes in HR+ cancer cells [12, 13], but it remains unknown whether this function is maintained in human tumors *in vivo*. We therefore investigated chromatin accessibility differences in accordance with disease progression in our dataset. Remarkably, average chromatin accessibility levels within each signature differed across tumor stages (Fig. 3A-C). Most notably, signature 2 regions, which are enriched for FOX motifs (Fig. 2A), displayed the strongest positive relationship, with progressively greater accessibility associating with increasing severity of disease (Fig. 3C & S3C). Additionally, accessibility levels across all FOX motifs, irrespective of genomic location, were positively correlated with tumor stage (Fig. 3D, E, S3D), despite no apparent differences in *FOXA1* gene expression levels between tumors of different stages (Fig. 3F).

These results indicate that chromatin accessibility at FOX factor binding motifs increases with tumor progression, and increased FOXA1 binding may underlie the chromatin patterns distinguishing group 2 from other tumor samples. To further investigate the relationship between FOX motif accessibility and cancer progression, we re-analyzed recently published ATAC-Seq data from Basal-L TNBC cell lines [34] and found that accessibility at signature 2 and FOX motifs sites was indeed higher in metastatic cells (late-stage) compared with non-metastatic (early-stage) cells (Fig. S3E). These results are both consistent with prior reports (mouse and *in vitro*), and are indicative of a molecular axis whereby FOXA1 binding increases in more advanced tumors independent of changes in FOXA1 expression levels, suggesting that additional factors are involved.

### *ANP32E* levels are associated with accessibility at FOX motifs and with tumor programming

**Fig. 4:**
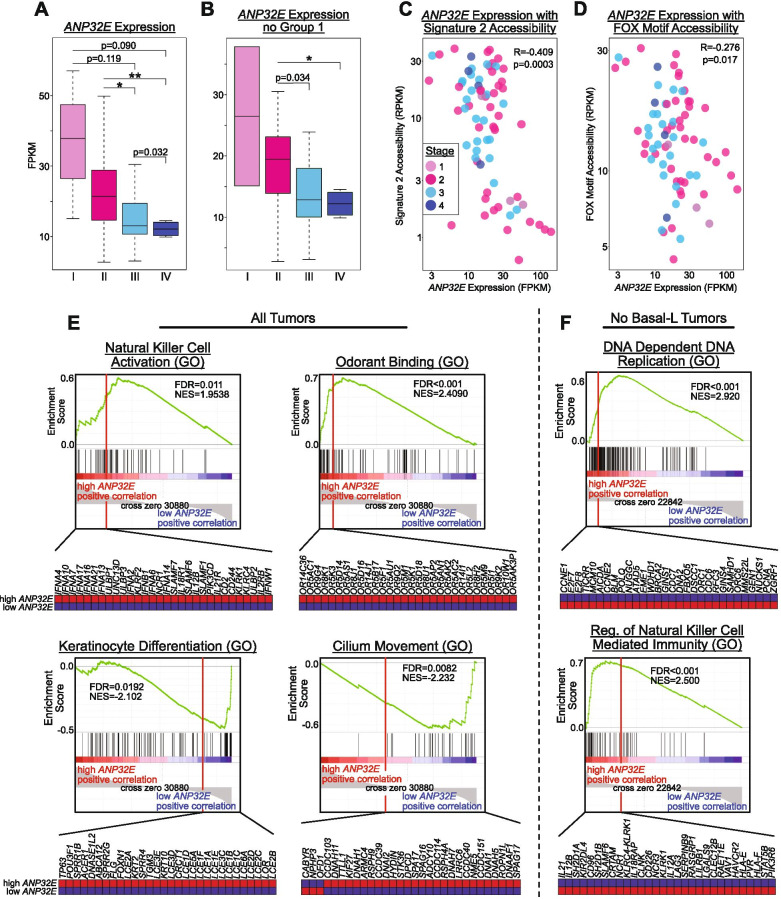
*ANP32E* Expression Levels Associate with FOX Motif Accessibility and Tumor Stage. A-B) Boxplots comparing *ANP32E* expression by tumor stage, both in all tumors with available stage data (n=73) (**A**) and in only tumors from groups 2 and 3 (n=59) (**B**), indicating that late-stage tumors have significantly lower expression of *ANP32E*. P-values obtained from one-tailed parametric t-tests. * is p<0.01, ** is p<0.001, *** is p<0.0001. C-D) Scatterplots showing correlation of *ANP32E* expression with tumor's accessibility in signature 2 regions (**C**) and with tumor's average accessibility in FOX motifs across all accessible peak regions (n=96280) (**D**), with tumors colored by stage. R denotes Pearson correlation coefficient; p-values from Pearson's product moment correlation coefficient. E-F) GSEA plots depicting gene ontology associations with high and low *ANP32E* expression levels for all tumors with RNA-seq data in the TCGA-BRCA project (n=1222) (**E**) and for all tumors excluding Basal-L (n=1110) (**F**). FDR values and normalized enrichment scores (NES) obtained within GSEA

The above data indicate that accessibility of FOX motifs is generally associated with tumor stage, but we find little evidence for differences in *FOXA1* (or *ESR1*) expression levels between tumors of different stages. We next investigated whether additional factors may contribute to the observed accessibility differences at FOX binding motifs. Prior studies of HR+ breast cancer cells have demonstrated that the function of FOXA1 is impacted by the local enrichment of the histone variant H2A.Z [48, 49]. H2A.Z accumulates at estrogen response elements that are bound by FOXA1 and loss of H2A.Z impairs both FOXA1 binding and polymerase recruitment. ANP32E is a chromatin chaperone that regulates the genomic localization of H2A.Z to control locus-specific chromatin state dynamics [14]. In recent work, we showed that ANP32E antagonizes H2A.Z installation, such that ANP32E loss causes a global increased H2A.Z enrichment, heightened chromatin accessibility and amplified transcription factor binding at open sites, in cultured mouse fibroblasts [14]. ANP32E may function similarly in breast tumors, influencing the binding of key oncogenic transcription factors, such as FOXA1. Therefore, we investigated the relationship between *ANP32E* expression, chromatin accessibility, and tumor characteristics across the chromatin-defined tumor groups (Fig. S3F). Consistent with a prior report looking at protein levels [15], we found *ANP32E* mRNA to be significantly higher in Basal-L tumors (within group 1) than the other PAM50 subtypes (Fig. S3F & S3G). Moreover, the levels of *ANP32E* expression tended to stratify tumors by stage, wherein early-stage (I, II) tumors had the highest levels of *ANP32E* expression and late-stage (III, IV) tumors had the lowest levels (Fig. 4A & S3H). This association was maintained even when group 1 tumors were excluded (Fig. 4B & S3I), indicating that *ANP32E* levels may be functionally involved in cancer progression independent of tumor subtype.

We next evaluated the relationship between *ANP32E* expression and accessibility, and found that accessibility at signature 2 regions (Fig. 4C) and all accessible FOX motifs (Fig. 4D) were significantly anticorrelated with levels of *ANP32E* expression across all tumors (signature 2: R= -0.409, p=0.0003; FOX motifs: R= -0.276, p=0.017), suggesting that ANP32E may function as a negative regulator of chromatin accessibility at these sites. Indeed, CUT&Tag experiments revealed that ANP32E was localized at sites of high H2A.Z enrichment which lacked FOXA1, and FOXA1 resided primarily at sites with moderate to low H2A.Z levels and lesser enrichment for ANP32E (Fig. S4A). Additionally, signature 2 regions had the highest levels of H2A.Z in MCF-7 cells (Fig. S4B).

Based on our findings that reduced *ANP32E* expression levels associated with tumor stage progression, perhaps through regulation of FOXA1 binding, we next sought to determine the relationship between *ANP32E* expression and tumor phenotype, using the tumor transcriptome as a read-out. GSEA analysis revealed that high *ANP32E* expression was associated with increased expression of genes involved in the immune response (Fig. 4E) and to a lesser extent DNA replication (Fig. S5A). Consistent with this idea, *KI67* expression, a marker of cellular proliferation [50], was highest in group 1 tumors (representing all Basal-L and most TNBC tumors) (Fig. S5B), and *ANP32E* and *KI67* levels were positively correlated across all samples analyzed, but not after removing group 1 (Basal-L) tumors (Fig. S5C). Conversely, low *ANP32E* expression was associated with increased expression of genes involved in separate developmental processes (eg. ‘Keratinocyte Differentiation’ and ‘Cilium Movement’). To test whether the observed gene expression associations were driven by differences between Basal-L and non-Basal-L tumors, we repeated these analyses exclusively assaying tumors classified as non-Basal-L (see methods). Here again, GSEA results indicated that high *ANP32E* expression was associated with genes involved in DNA replication and immune response (Fig. 4F & S5D), indicating that *ANP32E* expression differences are indeed able to stratify patients in accordance with differences in cellular programming, independent of tumor subtype.

Taken together, these results suggest that ANP32E may generally function to restrict chromatin changes at the beginning stages of tumor development, and loss of ANP32E promotes tumor progression by enabling more aggressive cancers. In this regard ANP32E may act to ‘lock in’ a defined chromatin state, and when tumor cells transition to later stages of cancer progression, ANP32E becomes downregulated, leading to increased chromatin accessibility at a defined set of gene regulatory regions, including sites where H3K27ac and H2A.Z are enriched, enhancer elements, and FOXA1 binding sites.

## Discussion

We set out to investigate how differences in chromatin state across separate breast tumors coincided with unique characteristics of cancer biology, and to investigate whether differences in chromatin patterns could provide insight into new cancer mechanisms. To test whether chromatin accessibility patterns differed in a biologically meaningful manner, we took an unsupervised approach, using a dimensional reduction method (UMAP) to group tumors based only on chromatin differences. With this approach, 74 breast cancer tumors were grouped into three distinct UMAP categories. Supporting the validity of our UMAP approach, we found that differences in chromatin patterns associated with several known breast cancer features, including IHC marker status (Fig. S1C), PAM50 subtype classification (Fig. S1D), and histological classification (Fig. S2G). We also uncovered several novel chromatin associations. For example, our UMAP analysis indicated that 6 HR+/HER2- tumors were more similar to TNBC tumors (Fig. 1A), and these tumors were distinct from other HR+/HER2- tumors. Further characterization revealed that these 6 samples, along with TNBC samples, were classified as Basal-L, suggesting that the chromatin state of Basal-L tumors drove the UMAP segregation patterns. Differences in tumor heterogeneity may contribute to these differences in grouping and IHC status. For example, HR+/HER2- tumors with non-uniform IHC staining may be more similar to TNBC tumors when considered in aggregate than homogeneously stained HR+/HER2- tumors. Another interesting possibility is that a subset of HR+/HER2- tumors may be mechanistically more similar to TNBC-like tumors, perhaps explaining why some HR+/HER2- tumors are resistant to hormone therapies [51]. These results highlight the potential use of chromatin accessibility measurements as a diagnostic tool, possibly enabling further subtyping of breast cancer tumors. For example, measurements of chromatin accessibility may allow for better ILC subtyping within Lum-A tumors, as well as Basal-L tumors within HER2-enriched subtypes. Further chromatin accessibility studies of larger datasets are necessary for defining these potential subtypes, and for comparing classifications with current subtyping methods. More comprehensive and longitudinal studies of breast cancer, measuring chromatin state changes along with IHC status and gene expression profiling, will help in establishing how chromatin differences account for the observed UMAP grouping. In the future, additional diagnostic tests of HR+/HER2- tumors may be necessary to assess intrinsic cell-type of origin, potentially strengthening predictions of therapy response.

We also found chromatin differences occurred in a subset of Lum-A tumors, which appeared to have chromatin patterns more similar to non-Lum-A tumors within UMAP group 2 (including Lum-B and HER2-enriched tumors). This subset had reduced expression for genes involved in immune response (Fig. 1B) and reduced accessibility at regions proximal to metabolism genes (Fig. S2F), despite no measurable difference in expression for typical breast cancer markers, such as *PR*, *ESR1*, and *ERBB2* (Fig. S2C). We observed similar patterns for lobular tumors, which also segregated into two classes (Fig. S2G). Previous studies examining differences in Lum-A carcinomas found that pathways similar to those active in group 3 tumors were also active in ILC (as compared with ductal carcinoma), including immune-related and metabolic pathways [47]. In this context, our results suggest that group 3 may represent invasive carcinomas, similar to those described previously [45–47]. In prior studies, phenotypic differences for invasive carcinomas associated with mutational status (e.g. *CDH1* – Fig. S1E), but we found this class of tumors segregated in a manner dependent on chromatin accessibility differences – as many tumors within group 3 lacked key mutations associated with ILC. Accordingly, prior studies found that ILC tumors had decreased FOXA1 activity (based on measurements of gene expression and mutation frequency) [45], and in our study, we found lower chromatin accessibility levels at FOXA1 binding sites in group 3 tumors, which we presume to be similar to the ILC subtype (Fig. 2B). In sum, our results support a model where loss of FOXA1 activity (and/or subsequent loss of DNA binding) in luminal tumors distinguishes ILC-like from other HR+ tumors (presumably occurring within UMAP group 2).

**Fig. 5:**
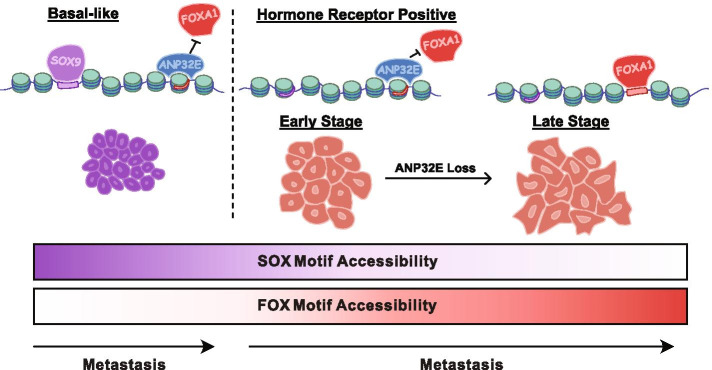
Model displaying the association of *ANP32E* expression levels with multiple characteristics of breast cancer. In Basal-L tumors, arising from a multipotent progenitor, we find that ANP32E may 'lock-in' a pattern of accessible chromatin favoring SOX9 binding, proliferation and self-renewal. Alternatively, in HR+ breast tumors arising from a more differentiated luminal progenitor, ANP32E restricts FOXA1 binding such that luminal identify and hormone responsiveness is maintained. The loss of ANP32E may therefore increase FOXA1 binding, relax cellular programming and increase the metastatic potential of the tumor

We found the tumors within UMAP group 2 to be particularly interesting, as several distinct cancer subtypes grouped together, indicating that they had quite similar chromatin accessibility patterns despite differences in clinical classifications. Interestingly, FOX motifs were enriched within the genomic loci where accessibility differences occurred (Fig. 2A & B) and these loci were located distal from gene promoters (Fig. 1E). In MCF-7 breast cancer cells, these regions are bound by FOXA1 and ER, and enriched for H2A.Z and H3K27ac (Fig. 2D and S4B), suggesting that they may function as enhancer elements in HR+ breast cancer tumors. Prior studies have demonstrated that H2A.Z levels at ER binding sites facilitates enhancer activation and FOXA1 binding in this type of (HR+) breast cancer cell [48, 49]. We and others previously demonstrated that H2A.Z is a negative regulator of DNA methylation [52–54], and accordingly, lower DNA methylation levels are known to occur at enhancers bound by FOXA1 and ER in luminal tumors (compared with basal tumors) [55]. Additionally, increased FOXA1 activity has been shown to function in the activation of pro-metastatic cellular programming [12]. Taken together, these results suggest that increased H2A.Z levels at enhancers in luminal tumors may promote increased accessibility, improved FOXA1 binding, and amplified enhancer activity, potentially driving tumors toward a more metastatic cellular program without obvious changes in FOXA1 expression levels (Fig. 5).

The histone chaperone ANP32E has previously been shown to control H2A.Z levels at thousands of vertebrate gene regulatory regions, including enhancers [14, 52, 56, 57]. We previously found that ANP32E functions in mouse cells to control genome-wide chromatin accessibility through regulation of H2A.Z patterns [14]. Based on this mechanism, differences in ANP32E levels among breast tumors may lead to differences in H2A.Z enrichment, causing chromatin accessibility differences, and ultimately impacting transcription factor binding events. Similar mechanisms have recently been described during tumorigenesis of uterine leiomyomas, wherein epigenetic instability due to H2A.Z depletion leads to chromatin accessibility and gene expression dysregulation, particularly at genes involved in hormone signaling [58]. In the context of this study, we do indeed find that *ANP32E* expression levels differ among tumors, and these differences are anticorrelated with chromatin accessibility at FOX factor binding sites. Interestingly, accessibility at these same sites tends to increase in later-stage tumors (stage III, IV), compared with earlier stages (stage I, II), suggesting that selective opening of signature 2 regions (and FOX binding in particular) may function to promote tumor progression. In this regard, ANP32E levels in HR+ tumors may specifically restrict chromatin accessibility at FOX factor motifs (Fig. 5). Additional mechanistic studies of ANP32E, H2A.Z, and their role in FOX factor binding in the context of HR+ breast cancer are necessary to fully investigate this possibility.

It is important to note that our study investigated accessibility data from primary tumor samples only. In this context, our ability to identify significant correlations between stage at resection and chromatin accessibility suggests that changes in the chromatin state of the primary tumor may precede, and/or be predictive of, the propensity for tumor progression and/or metastatic spread. We therefore propose a model in which ANP32E has two separate functions in breast cancer, depending on tumor subtype or the differentiation state of the cell of origin. In Basal-L/TNBC tumors, largely believed to arise from a more stem-like multipotent progenitor, high levels of ANP32E ‘lock-in’ a pattern of accessible chromatin that favors proliferation and self-renewal, while in HR+ breast tumors, arising in a more differentiated luminal progenitor, ANP32E supports the maintenance of luminal identity and hormone responsiveness by restricting FOXA1 binding at estrogen response elements (Fig. 5). In this latter setting, the loss of ANP32E expression may lead to increased FOXA1 binding, relaxation of cellular programming, and progression to a hormone-resistant state. Indeed, factors affecting the balance of ER and FOXA1 binding to estrogen response elements, such as forced overexpression of FOXA1, may promote expression of genes involved in metastasis and endocrine-resistant breast cancers [12]. The mechanistic function of ANP32E remains only partially understood, and phenotypes have not been observed in mice lacking ANP32E. Our studies suggest that additional phenotypes may arise under disease conditions, upon exposure to external stressors, or with concomitant loss of additional ANP32 factors. Evidently, future studies addressing the role of ANP32E, H2A.Z, and their role in FOX factor binding, are necessary to fully establish the function of ANP32E in carcinogenesis.

## Conclusions

We applied an unsupervised machine learning-based platform (UMAP) to investigate chromatin states within 74 human breast cancer tumors. This methodology enabled us to identify chromatin differences with no prior knowledge of tumor subtype or disease outcome. While fairly common for single-cell genomics studies, approaches such as ours have only recently been applied to studies of carcinogenesis. In principle, such methods could be used extensively to deconvolute the high dimensional datasets that are publicly available within the TGCA repository. In this regard, our study sets a precedent for future computational studies of tumor biology.

We found that 3 major chromatin states occurred within breast cancer tumor samples. One class represented Basal-L tumors, while the other two occurred nearly independent of tumor subtype classification. Chromatin accessibility levels at FOXA1 binding sites both segregated tumors within these latter two classes, and correlated with disease progression, suggesting that FOXA1 may function broadly across breast cancer tumors to promote metastasis. Rather than finding differences in *FOXA1* expression, we found that expression levels for the chromatin regulatory factor *ANP32E* were anticorrelated with accessibility at FOXA1 binding sites. *ANP32E* expression was lowest in late-stage hormone positive tumors, and highest in early-stage TNBC tumors, suggesting that ANP32E levels may affect cellular programming through regulation of FOXA1 binding. Finally, our ability to identify such correlations despite exclusively examining primary tumors (with no measurements of distal metastases) suggests that ANP32E reduction and chromatin state changes likely occur in the primary tumor prior to metastatic spread.

## Supplementary Information

**Additional file 1: Figure 1 supplement.** A-B) For patients whom tumors were obtained from, boxplot showing age at diagnosis (A) and stacked barplot displaying racial identity (B). Significance values obtained within cBioPortal with Kruskal-Wallis test. C-D) Individual pie charts depicting groups of tumors based on IHC subtypes (C) and PAM50 subtypes (D), indicating tumor groups distinguish breast cancer subtypes. E) UMAP plots colored by tumor's mutation status for commonly mutated genes in the TCGA-BRCA project. F) Boxplots comparing gene expressions for PAM50 genes by tumor group. G-H) Scatterplots depicting genes found to have higher or lower expression in HR+/HER2- tumors in group 1 (n=6) compared to rest (n=40) (G) and in Luminal-A tumors in group 3 (n=8) compared to group 2 (n=17) (H). P-values in F obtained from one-tailed parametric t-tests. * is p<0.01, ** is p<0.001, *** is p<0.0001. **Figure 2 Supplement.** A) Bar chart depicting significance of gene ontology results from Enrichr, investigating genes found to have higher and lower expression in HR+/HER2- tumors in group 1 (n=6) compared to rest (n=40). Adjusted p-values obtained within Enrichr. B-C) Boxplots comparing gene expression of hormone receptors in TNBC tumors (n=7) and HR+/HER2- tumors separated into group 1 (n=6) and rest (n=40) (B) and in Luminal-A tumors separated into all (n=25), group 2 (n=17) and group 3 (n=8) (C). D) Bar charts depicting significance of gene ontology results from GREAT, investigating genes nearby (<1000 kb) regions found to have higher and lower accessibility in group 3 Luminal-A tumors compared to group 2. E) Scatterplot depicting regions found to have higher or lower accessibility in Luminal-A tumors in group 3 (n=8) compared to group 2 (n=17). F) Bar charts depicting significance of gene ontology results from GREAT, investigating genes nearby (<1000 kb) regions found to have higher and lower accessibility in group 3 Luminal-A tumors compared to group 2. G) Stacked barplot showing the distribution of histological subtypes between each tumor group. P-value obtained from Chi-squared test within cBioPortal. FDR q-values in D and F obtained within GREAT. P-values in B-C obtained from one-tailed parametric t-tests. * is p<0.01, ** is p<0.001, *** is p<0.0001. **Figure 3 supplement.** A) Boxplots comparing expression across tumor groups of interesting SOX and FOX factors from figure 2C. B) Boxplot comparing tumor's average accessibility at FOX motifs within accessible peak regions (n=96280) for Basal-L tumors (n=12) and Lum-A tumors within groups 2 (n=17) and 3 (n=8). C-D) Beeswarm plots comparing tumor's average accessibility in signature 2 regions (n=1314) (C) and tumor's average accessibility at FOX motifs within accessible peak regions (D) by tumor stage. E) Profile plots showing accessibility at FOX motifs and signature regions in TNBC cell-lines, ignoring regions with no signal in the calculation of average accessibility. MB-231 Par is a normal MB-231 cell-line, while MB-231 BrM and MB-231 LM are MB-231 cell-lines with high metastatic potential to brain and lung, respectively. F) Heatmap with tumors ordered by *ANP32E* expression and annotated by tumor group, PAM50 subtype and tumor stage. G) Boxplot comparing *ANP32E* expression of tumors by PAM50 subtype. H-I) Beeswarm plots comparing *ANP32E* expression by tumor stage for all tumors with available stage data (n=73) (H) and for only group 2 and 3 tumors (n=59) (I). P-values in A-D and G-I obtained with one-tailed parametric t-tests. * is p<0.01, ** is p<0.001, *** is p<0.0001. **Figure 4 Supplement.** A) Heatmaps showing binding of FOXA1, ANP32E and H2AZ at regions classified as either ANP32E or FOXA1 binding peaks in MCF-7 cells (n=35916). Regions sorted by average enrichment of FOXA1. B) Profile plot showing binding of H2A.Z in MCF-7 cells within regions from signatures 1, 2 and 3. Data from ChIP-Seq of MCF-7 cells. **Figure 5 Supplement.** A) GSEA plots depicting gene ontology associations with high and low *ANP32E* expression levels for all tumors with RNA-seq data from the TCGA-BRCA project (n=1222). B) Boxplot of *KI67* expression across tumor groups. P-value from one-tailed parametric t-test. C) Scatterplot showing the association between *ANP32E* and *KI67* expression levels, with tumors colored by tumor group. R denotes Pearson correlation coefficient; p-values from Pearson's product moment correlation coefficient (done for all tumors, and all tumors excluding group 1). D) GSEA plots depicting gene ontology associations with high and low *ANP32E* expression levels for non-Basal-L tumors (n=1110). FDR and NES values in A and D obtained within GSEA

## Data Availability

The ATAC-Seq datasets of chromatin accessibility, the gene expression datasets, tumor stage data, and IHC subtype data are all available from TCGA-BRCA project in the NCI’s GDC [16] (https://portal.gdc.cancer.gov/). PAM50 subtype [19], histological subtype [20], and general patient demographics [20] data are available in cBioPortal [21, 22] (https://www.cbioportal.org/). ChIP-Seq data from the MCF-7 cell line is available from ENCODE [32, 33] (https://www.encodeproject.org/). ENCODE accession numbers: for FOXA1 (ENCSR126YEB), H3K27ac (ENCSR752UOD), H2A.Z (ENCSR057MWG) and ER (ENCSR463GOT). BigWig files of log2FC over control are available from the ENCODE portal with the following identifiers: ENCFF795BHZ (FOXA1), ENCFF063VLJ (H3K27ac), ENCFF589PLM (H2A.Z), and ENCFF237WTX (ER). ATAC-Seq data from TNBC cell-lines was acquired using accession number GSE129646 )[34]. CUT&Tag data measuring ANP32E in MCF7 cells can be found using GEO accession number GSE188942.

## Abbreviations

TCGA: The Cancer Genome Atlas
NCI: National Cancer Institute
GDC: Genomic Data Commons
log2FC: log2 fold change
ATAC-Seq: assay for transposase-accessible chromatin followed by sequencing
UMAP: Uniform Manifold Approximation and Projection
ER: Estrogen Receptor
PR: Progesterone Receptor
HR: hormone receptor
Lum-A: Luminal A
Lum-B: Luminal B
Basal-L: Basal-like
IHC: immunohistochemistry
GO: gene ontology
ILC: invasive lobular carcinoma
MB-231 Par: normal MB-231 cell-line
MB-231 BrM: MB-231 cell-line with high metastatic potential to brain
MB-231 LM: MB-231 cell-line with high metastatic potential to lung
