# Supplementary material for: Subtype-Independent ANP32E Reduction During Breast Cancer Progression in Accordance with Chromatin Relaxation
Source: BMC Cancer. 2021 Dec 18;21:1342. doi: 10.1186/s12885-021-09077-9 (PMC8684129; doi:10.1186/s12885-021-09077-9)
Supplement: Supplementary file 1 — Additional file 1: Figure 1 supplement. A-B) For patients whom tumors were obtained from, boxplot showing age at diagnosis (A) and stacked barplot displaying racial identity (B). Significance values obtained within cBioPortal with Kruskal-Wallis test. C-D) Individual pie charts depicting groups of tumors based on IHC subtypes (C) and PAM50 subtypes (D), indicating tumor groups distinguish breast cancer subtypes. E) UMAP plots colored by tumor's mutation status for commonly mutated genes in the TCGA-BRCA project. F) Boxplots comparing gene expressions for PAM50 genes by tumor group. G-H) Scatterplots depicting genes found to have higher or lower expression in HR+/HER2- tumors in group 1 (n=6) compared to rest (n=40) (G) and in Luminal-A tumors in group 3 (n=8) compared to group 2 (n=17) (H). P-values in F obtained from one-tailed parametric t-tests. * is p<0.01, ** is p<0.001, *** is p<0.0001. Figure 2 Supplement. A) Bar chart depicting significance of gene ontology results from Enrichr, investigating genes found to have higher and lower expression in HR+/HER2- tumors in group 1 (n=6) compared to rest (n=40). Adjusted p-values obtained within Enrichr. B-C) Boxplots comparing gene expression of hormone receptors in TNBC tumors (n=7) and HR+/HER2- tumors separated into group 1 (n=6) and rest (n=40) (B) and in Luminal-A tumors separated into all (n=25), group 2 (n=17) and group 3 (n=8) (C). D) Bar charts depicting significance of gene ontology results from GREAT, investigating genes nearby (<1000 kb) regions found to have higher and lower accessibility in group 3 Luminal-A tumors compared to group 2. E) Scatterplot depicting regions found to have higher or lower accessibility in Luminal-A tumors in group 3 (n=8) compared to group 2 (n=17). F) Bar charts depicting significance of gene ontology results from GREAT, investigating genes nearby (<1000 kb) regions found to have higher and lower accessibility in group 3 Luminal-A tumors compared to group 2. G) Stacked barplot s [file 12885_2021_9077_MOESM1_ESM.pdf]

Supp. Fig. 1

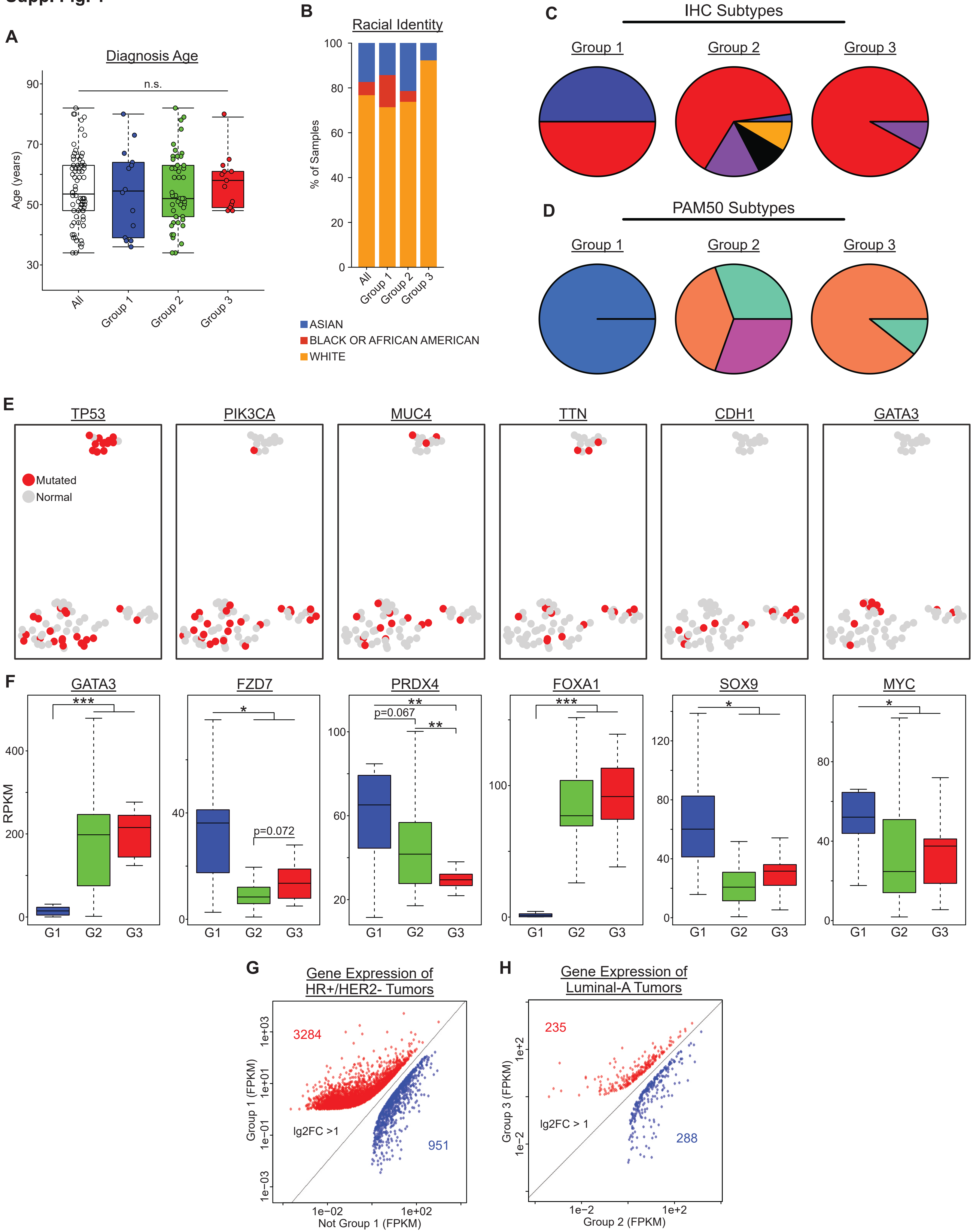

A
Diagnosis Age
n.s.
Age (years)
90
70
50
30
All
Group 1
Group 2
Group 3
B
Racial Identity
% of Samples
100
80
60
40
20
0
All
Group 1
Group 2
Group 3
ASIAN
BLACK OR AFRICAN AMERICAN
WHITE
C
IHC Subtypes
Group 1
Group 2
Group 3
D
PAM50 Subtypes
Group 1
Group 2
Group 3
E
TP53
PIK3CA
MUC4
TTN
CDH1
GATA3
Mutated
Normal
F
GATA3
FZD7
PRDX4
FOXA1
SOX9
MYC
RPKM
***
*
p=0.072
**
p=0.067
**
***
*
*
G1
G2
G3
G
Gene Expression of
HR+/HER2- Tumors
3284
951
lg2FC >1
Group 1 (FPKM)
Not Group 1 (FPKM)
1e+03
1e+01
1e−01
1e−03
1e−02
1e+02
H
Gene Expression of
Luminal-A Tumors
235
288
lg2FC > 1
Group 3 (FPKM)
Group 2 (FPKM)
1e+2
1e−2

Supp. Fig. 2

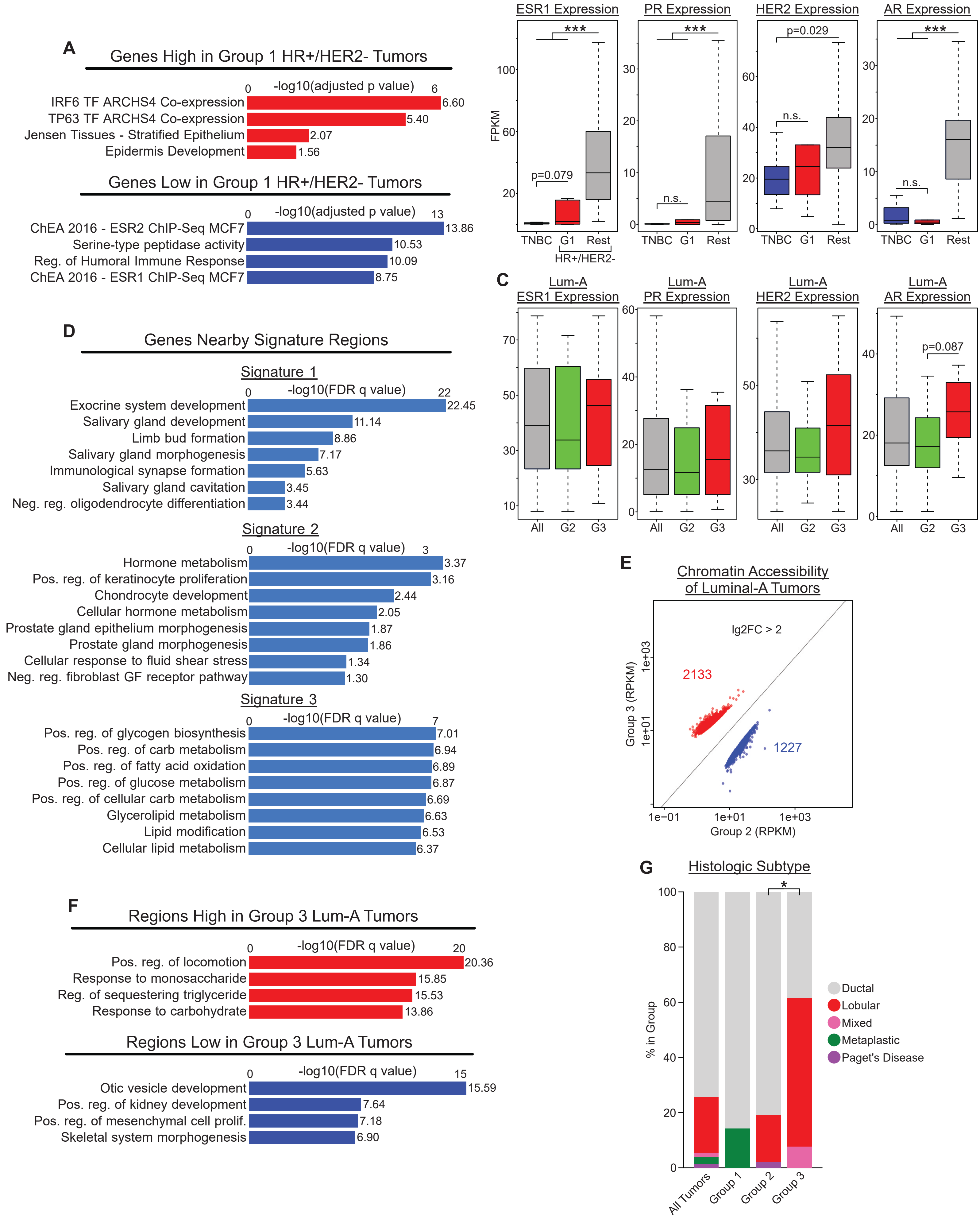
A
Genes High in Group 1 HR+/HER2- Tumors
-log10(adjusted p value)
0
6
IRF6 TF ARCHS4 Co-expression 6.60
TP63 TF ARCHS4 Co-expression 5.40
Jensen Tissues - Stratified Epithelium 2.07
Epidermis Development 1.56
Genes Low in Group 1 HR+/HER2- Tumors
-log10(adjusted p value)
0
13
ChEA 2016 - ESR2 ChIP-Seq MCF7 13.86
Serine-type peptidase activity 10.53
Reg. of Humoral Immune Response 10.09
ChEA 2016 - ESR1 ChIP-Seq MCF7 8.75
B
ESR1 Expression
PR Expression
HER2 Expression
AR Expression
FPKM
***
p=0.079
p=0.029
n.s.
TNBC
G1
Rest
HR+/HER2-
C
Lum-A ESR1 Expression
Lum-A PR Expression
Lum-A HER2 Expression
Lum-A AR Expression
p=0.087
All
G2
G3
D
Genes Nearby Signature Regions
Signature 1
-log10(FDR q value)
0
22
Exocrine system development 22.45
Salivary gland development 11.14
Limb bud formation 8.86
Salivary gland morphogenesis 7.17
Immunological synapse formation 5.63
Salivary gland cavitation 3.45
Neg. reg. oligodendrocyte differentiation 3.44
Signature 2
-log10(FDR q value)
0
3
Hormone metabolism 3.37
Pos. reg. of keratinocyte proliferation 3.16
Chondrocyte development 2.44
Cellular hormone metabolism 2.05
Prostate gland epithelium morphogenesis 1.87
Prostate gland morphogenesis 1.86
Cellular response to fluid shear stress 1.34
Neg. reg. fibroblast GF receptor pathway 1.30
Signature 3
-log10(FDR q value)
0
7
Pos. reg. of glycogen biosynthesis 7.01
Pos. reg. of carb metabolism 6.94
Pos. reg. of fatty acid oxidation 6.89
Pos. reg. of glucose metabolism 6.87
Pos. reg. of cellular carb metabolism 6.69
Glycerolipid metabolism 6.63
Lipid modification 6.53
Cellular lipid metabolism 6.37
E
Chromatin Accessibility of Luminal-A Tumors
lg2FC > 2
2133
1227
Group 3 (RPKM)
Group 2 (RPKM)
1e−01
1e+01
1e+03
F
Regions High in Group 3 Lum-A Tumors
-log10(FDR q value)
0
20
Pos. reg. of locomotion 20.36
Response to monosaccharide 15.85
Reg. of sequestering triglyceride 15.53
Response to carbohydrate 13.86
Regions Low in Group 3 Lum-A Tumors
-log10(FDR q value)
0
15
Otic vesicle development 15.59
Pos. reg. of kidney development 7.64
Pos. reg. of mesenchymal cell prolif. 7.18
Skeletal system morphogenesis 6.90
G
Histologic Subtype
% in Group
*
Ductal
Lobular
Mixed
Metaplastic
Paget's Disease
All Tumors
Group 1
Group 2
Group 3

Supp. Fig. 3

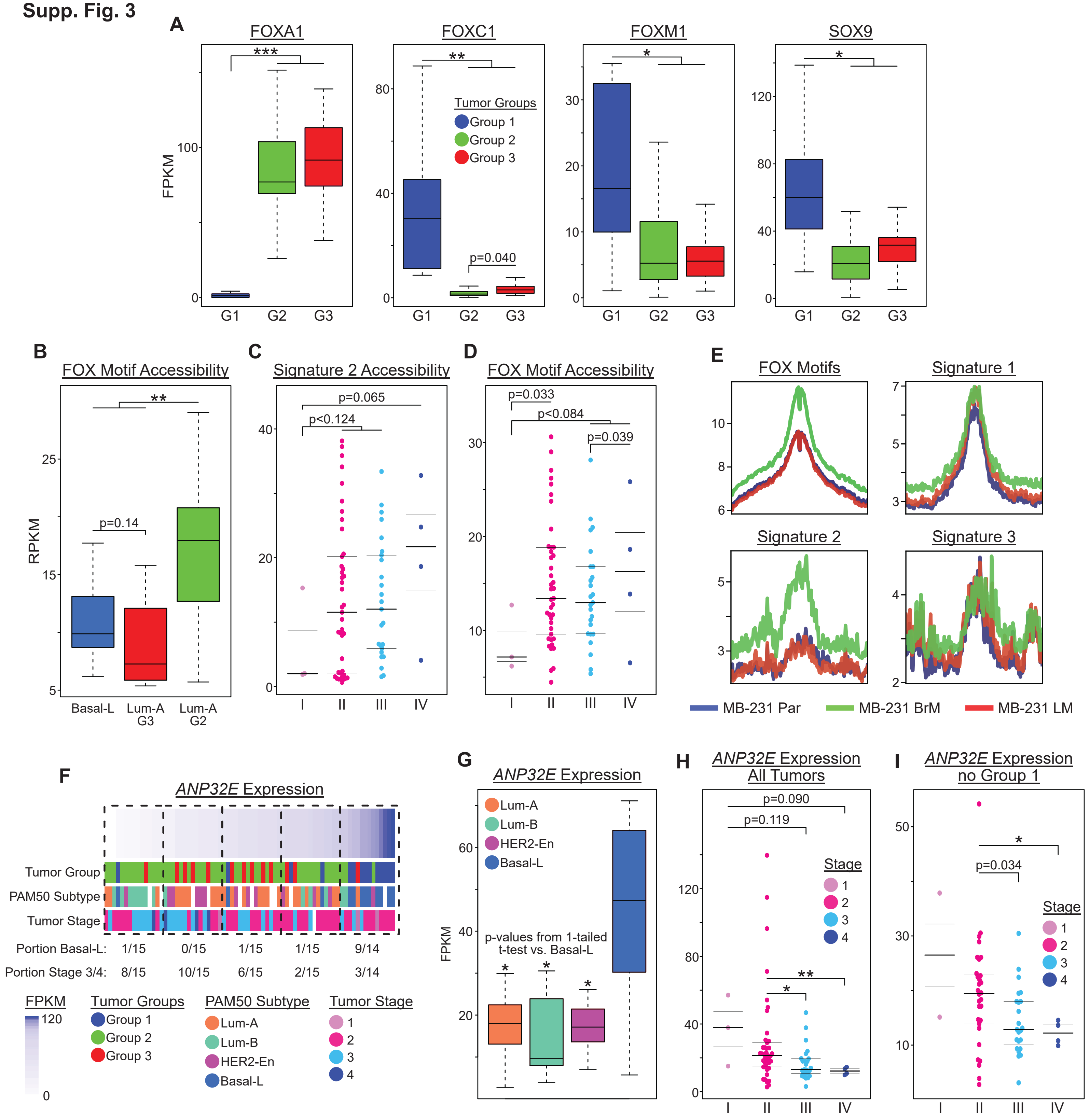
A
FOXA1
FOXC1
FOXM1
SOX9
FPKM
Tumor Groups
Group 1
Group 2
Group 3
p=0.040
G1 G2 G3
B
FOX Motif Accessibility
RPKM
p=0.14
Basal-L
Lum-A G3
Lum-A G2
C
Signature 2 Accessibility
p=0.065
p<0.124
I II III IV
D
FOX Motif Accessibility
p=0.033
p<0.084
p=0.039
E
FOX Motifs
Signature 1
Signature 2
Signature 3
MB-231 Par
MB-231 BrM
MB-231 LM
F
ANP32E Expression
Tumor Group
PAM50 Subtype
Tumor Stage
Portion Basal-L: 1/15 0/15 1/15 1/15 9/14
Portion Stage 3/4: 8/15 10/15 6/15 2/15 3/14
FPKM
120
0
Tumor Groups
Group 1
Group 2
Group 3
PAM50 Subtype
Lum-A
Lum-B
HER2-En
Basal-L
Tumor Stage
1
2
3
4
G
ANP32E Expression
Lum-A
Lum-B
HER2-En
Basal-L
p-values from 1-tailed t-test vs. Basal-L
FPKM
H
ANP32E Expression All Tumors
p=0.090
p=0.119
Stage
I
ANP32E Expression no Group 1
p=0.034
Stage

Supp. Fig. 4

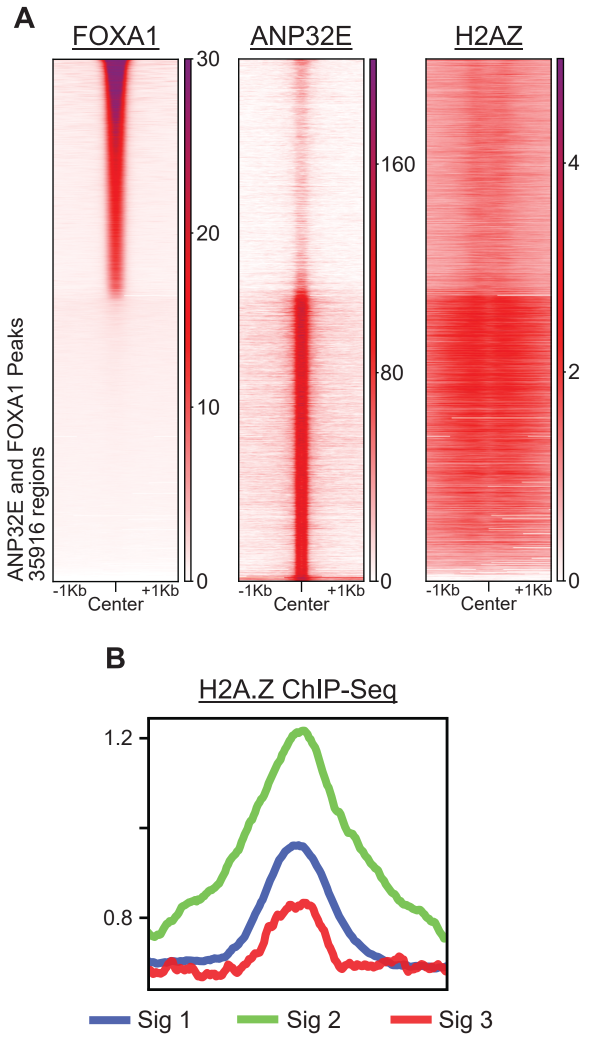
A
FOXA1
ANP32E
H2AZ
30
20
10
0
160
80
0
4
2
0
ANP32E and FOXA1 Peaks
35916 regions
-1Kb
Center
+1Kb
B
H2A.Z ChIP-Seq
1.2
0.8
Sig 1
Sig 2
Sig 3

Supp. Fig. 5

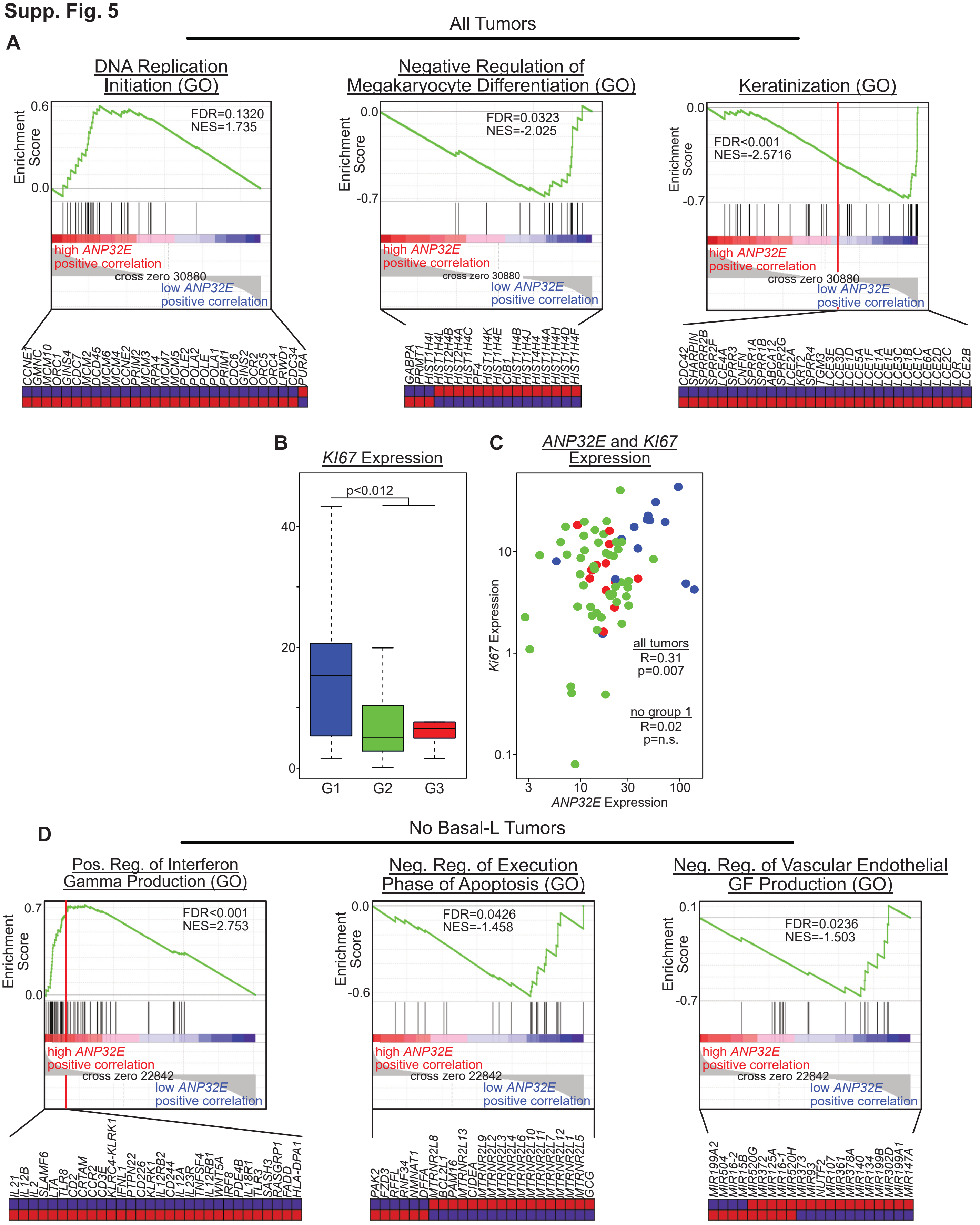

All Tumors
A
DNA Replication Initiation (GO)
FDR=0.1320
NES=1.735
Negative Regulation of Megakaryocyte Differentiation (GO)
FDR=0.0323
NES=-2.025
Keratinization (GO)
FDR<0.001
NES=-2.5716
Enrichment Score
high ANP32E positive correlation
low ANP32E positive correlation
cross zero 30880
B
KI67 Expression
p<0.012
G1
G2
G3
C
ANP32E and KI67 Expression
KI67 Expression
ANP32E Expression
all tumors
R=0.31
p=0.007
no group 1
R=0.02
p=n.s.
D
No Basal-L Tumors
Pos. Reg. of Interferon Gamma Production (GO)
FDR<0.001
NES=2.753
Neg. Reg. of Execution Phase of Apoptosis (GO)
FDR=0.0426
NES=-1.458
Neg. Reg. of Vascular Endothelial GF Production (GO)
FDR=0.0236
NES=-1.503
cross zero 22842
